# Hands-Free User Interface for VR Headsets Based on In Situ Facial Gesture Sensing

**DOI:** 10.3390/s20247206

**Published:** 2020-12-16

**Authors:** Jinhyuk Kim, Jaekwang Cha, Shiho Kim

**Affiliations:** Seamless Transportation Laboratory, School of Integrated Technology, Yonsei University, Incheon 21983, Korea; jinhyuk.kim@yonsei.ac.kr (J.K.); chajae42@yonsei.ac.kr (J.C.)

**Keywords:** head-mounted displays, sensing facial gestures, infrared sensors, hands-free user interfaces

## Abstract

The typical configuration of virtual reality (VR) devices consists of a head-mounted display (HMD) and handheld controllers. As such, these units have limited utility in tasks that require hand-free operation, such as in surgical operations or assembly works in cyberspace. We propose a user interface for a VR headset based on a wearer’s facial gestures for hands-free interaction, similar to a touch interface. By sensing and recognizing the expressions associated with the in situ intentional movements of a user’s facial muscles, we define a set of commands that combine predefined facial gestures with head movements. This is achieved by utilizing six pairs of infrared (IR) photocouplers positioned at the foam interface of an HMD. We demonstrate the usability and report on the user experience as well as the performance of the proposed command set using an experimental VR game without any additional controllers. We obtained more than 99% of recognition accuracy for each facial gesture throughout the three steps of experimental tests. The proposed input interface is a cost-effective and efficient solution that facilitates hands-free user operation of a VR headset using built-in infrared photocouplers positioned in the foam interface. The proposed system recognizes facial gestures and incorporates a hands-free user interface to HMD, which is similar to the touch-screen experience of a smartphone.

## 1. Introduction

In traditional input interfaces of personal devices, hand input is the mainstream approach. These include interfaces based on keyboards, mouse, and touch-screens. In line with this trend, VR devices using handheld controllers have also been popularized. However, since VR content is becoming diversified, the limitation of the handheld controller is revealed in operations that require freedom of the hands, for example, remote surgery that requires hands-on operations or an immersive VR game content that uses a separate input device such as a model gun. In these cases, an additional input method that enables a simple interface operation or command execution without using the hands would be useful.

In a previous study, it was shown that individuals prefer to avoid using their hands for simple interface operations. Tung and Ying-Chao et al. showed that in VR/augmented reality (AR) environments, users prefer nonhandheld, nontouch interactions over handheld interactions [1]. It is necessary to introduce a new hands-free user interface that provides convenience to users who have to use their hands to work in a VR environment.

Research on the input interface can be generally divided into touch interaction and touchless interaction, including gaze tracking, hand gestures, and voice recognition, which could also be referred to as hands-free. Lee et al. defined a hands-free interface as an input method that does not make use of the hands, and freehand is an input method that uses a glove-based hand interface [2]. In this report, we define a hands-free interface as a case where head-mounted display (HMD) users do not use their hands for interaction with the contents or HMD. With regard to the VR headset, typical hands-free input methods are based on voice recognition and gaze tracking. Human eye movements include various types of information such as facial expressions and user intention. Several researchers have investigated human eye movement using multimodal sensors, including eye or gaze trackers [3,4,5,6]. As shown in Table 1, some researchers have integrated gaze tracking into a near-eye viewing device [7], endoscope camera [8], or head-mounted display (HMD) environment [9] to create a hands-free input interface. Many different hand gesture interfaces have been proposed in literature. Due to the recent introduction of depth cameras, new approaches have been investigated to combine existing methods with depth cameras [10,11] or body motion [12]. Regarding voice recognition, researchers have investigated methods for reducing the error in mobiles in noisy environments [13] and their operation at a low memory utilization rate [14].

In terms of user interface of VR headsets, each hands-free method might have advantages and disadvantages because each method depends on different modality of sensor inputs. Unconstrained real-time gaze estimation relative to the head pose is the key technology of the gaze tracking method [9]. For hands-free interactions, the user must freely control cursor movements and switching between interaction events through gaze times or through a blinking action. Additional technical difficulty of the eye tracking method is that the relative position between camera and eyes varies from person to person when an individual person wears an HMD [9].

Head-mounted gesture-based interfaces or voice recognition provide a solution for natural interaction. However, gesture or voice interactions with the headset interface typically require users to perform predefined poses or actions that can be cumbersome and distracting while performing a task with two-handed manipulations. Among those hands-free methods, the proposed interface utilizes facial skin movement as an input modality. The proposed user input device utilizing facial skin movements is a unique method for VR headsets [16,17].

Given that the VR environment requires various input devices depending on the content, it is necessary to use input devices that are capable of performing complex tasks. For painting or playing musical instruments, the user must use handheld controllers or hand gestures. Moreover, to face the VR avatars and interact with them, the user’s gaze must be tracked. Voice recognition can also be used for a wide range of complex commands [18]. However, in terms of a simple pop-up menu operation or interface control, it is more efficient to use a method that can perform repetitive tasks with higher accuracy in a simple manner. By utilizing a low-cost auxiliary input method, it is possible to reduce the use of unnecessary computational resources and enhance usability and user convenience with extended capability.

We propose a facial gesture recognition system using the user’s natural winking and facial movements around the eyes as a new input that can be used in parallel with the existing input method. To read the user’s facial gestures, we introduce an HMD interface foam in which an IR sensor module consisting of an adjacent IR emitter and receiver are embedded. The IR sensor pairs are placed at six points on the human face that show the largest intensity change for a given facial gesture. The proposed approach has the following novelty: first, the sensor is invisible because it is embedded inside the foam interface; and second, it is a natural user interface that operates only with the user’s facial gesture without using any additional devices. Therefore, it is a hands-free as well as cost-effective solution for a VR headset controller. The proposed input interface reads the user’s intention based on the change in the IR intensity and defines a command set by mapping these changes one-to-one with facial gestures.

### Related Works

In this study, the user’s facial expression was detected by illuminating the skin with IR light. Instead of allowing the light to propagate through the air, the IR emitter was in direct contact with the skin. After scattering and attenuation by the facial tissue, the IR rays were detected by an adjacent IR receiver. By recording the intensity of the IR signal, the IR receiver can detect the degree of facial muscle movement. In previous research of [17], a prototype in which two IR sensor pairs were used to detect facial skin movement and recognize a user’s intentional wink was presented. Moreover, an input system and a facial expression recognition system based on the same principle have been previously reported [19,20,21]. Cha et al. measured the intensity change of the scattered IR intensity due to the contraction and relaxation of skin in four areas (the abdomen, cheek, forearm, and thigh areas), and expressed the results as a function of the distance between the sensors [22]. The results indicate that the propagation of IR rays into tissue can be measured for various parts of the body. Furthermore, sensor modules with laser diodes and IR cameras instead of IR sensor pairs have been proposed [16,19,20]. The IR propagation characteristics can be used in AR environments in which the skin and sensor are not in close contact [16]. Using an unsupervised deep neural network for an AR headset, the classifier exhibited a user-command recognition rate of 95.4%.

**Figure 1:**
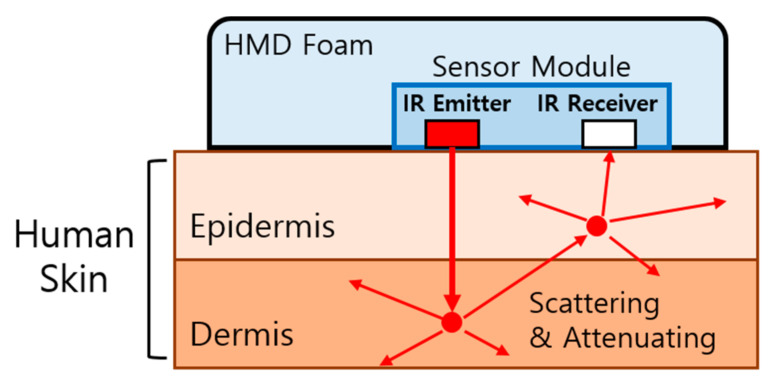
Principles of sensing: six sensor modules were used, each consisting of an IR emitter and receiver. Because the change in the scattered IR intensity due to the contraction and relaxation of the skin can be expressed as a function of the distance between the sensors [22], the degree of the skin contraction and relaxation can be determined based on the IR intensity; the distance between the IR sensors is fixed.

Although a device for facial expression recognition must use several sensors and IR cameras to detect movement of the entire face, in this study the number of sensors that can be attached to the interface foam of the HMD was limited to six. Cha et al. expressed the IR intensity as a function of the distance between the sensors [22]. As shown in Figure 1, the distance between the sensors in the HMD environment was fixed, and the degrees of contraction and relaxation of the skin could be measured by a sensor module composed of IR photocoupler.

## 2. Methods

In [18], only two pairs of sensors were used at the bottom of the HMD interface foam to detect the movements of the cheek muscles, which were classified using simple thresholds. In this study, the sensor pairs were placed in six specified areas of the face to monitor the movement of the muscles required for winking. This was done to identify the order in which the muscles move during a facial gesture. These six positions were theoretically determined according to the facial action coding system (FACS) standard [23]. They were also experimentally adjusted to produce the greatest changes in the sensor values for the HMD interface foam.

### 2.1. Apparatus

When a user makes a facial gesture, the tissue of the face contracts or relaxes depending on the muscle movement, which changes the intensity of the IR light that propagates through the tissue. Thus, by measuring the magnitude of the IR intensity at multiple sites, the facial gestures of the user can be determined. The proposed sensor should be placed at the positions with the greatest skin deformation during facial gestures. Therefore, the sensor pairs were placed in the regions of the cheek and eyebrow muscles (Orbicularis oculi), and the inner/outer brow muscles (Frontalis) [23,24]. Given that the muscle movement associated with facial gesture resembles winking, required muscle movement is hereafter defined as winking.

**Figure 2:**
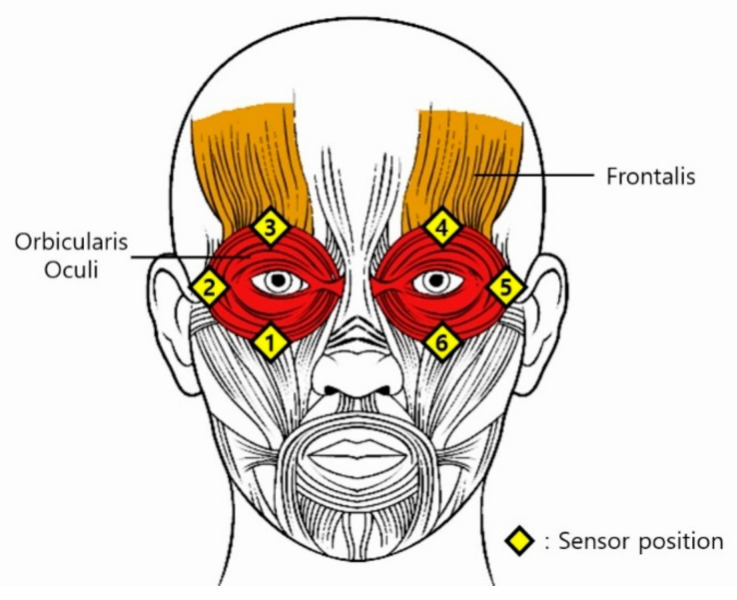
Location of Orbicularis oculi and Frontalis muscles. The Orbicularis oculi opens and closes the eyelid, whereas the Frontalis moves the eyebrow. The sensors are at the positions with the greatest contraction when an individual intentionally winks. Each number corresponds to the sensor number on the interface foam.

During winking, the muscles of the cheek contract, and consequently, the recorded values of the sensor at positions 1 and 6 specified in Figure 2 decrease. In contrast, given that the eyebrows and temples relax, the values of the sensors at positions 2, 3, 4, and 5 are expected to increase. This process creates friction between the skin and the sensor. The intended position of the sensor module is cut out from the interface foam, and the module is inserted such that it is flush with the interface surface. The embedded sensing module should be installed to minimize the friction between the skin and the sensor when the sensor is in close contact with the skin.

**Figure 3:**
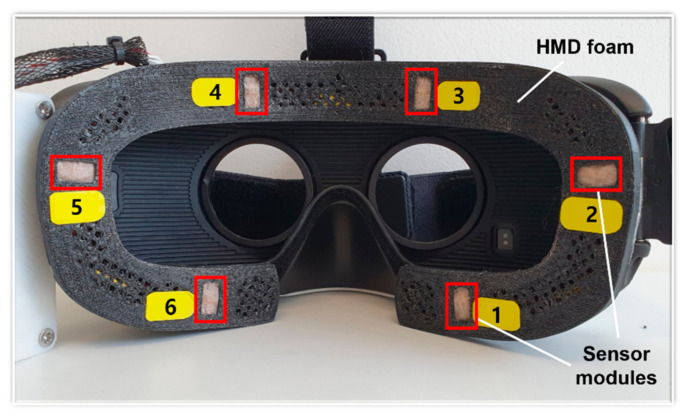
Fabricated prototype: each sensor module consists of adjacent IR emitters and receivers, and is installed on the inside of the foam interface. A part of the foam was scraped off to ensure that the sensors are in close contact with the skin.

We implemented IR photocouplers using Lumiled IR LEDs (wavelength of 850 nm and maximum power of 1050 mW) and a photodiode from Osram (SFH-2701). Given that the IR source emitted light directly onto the surface of the skin (particularly, close to the eyes, which could potentially affect the cornea and retina), LEDs with low intensities were selected in accordance with the IEC-62471 standard [25]. For the constructed prototype shown in Figure 3, the sensor modules are embedded in the interface foam of a Samsung Gear VR. However, the sensor modules can easily be implemented on most commercially available HMDs.

### 2.2. Command Set

The proposed hands-free user system consists of an interface foam equipped with six pairs of IR sensors to read facial gestures and an input command set that is mapped one-to-one with each gesture. It recognizes the user’s intention based on the sensing of facial gestures in situ, without any additional equipment except for built-in IR couplers inside the interface location of the HMD. Subsequently, it interprets the user’s facial gesture as a real-time command and uses it as an input.

The aim of the proposed user interface is to implement the functionality of the most essential and basic interfaces such as a conventional mouse or a touchpad in the standard HMD. The most basic commands commonly associated with these interfaces include clicking, double-clicking, dragging and dropping, and zooming in/out. These commands should be executable in a simple and repeatable manner. This is because the movement of the cursor is based on the orientation or motion of a gyroscope in an HMD. Given that a gyroscope is a commonly used device in headsets or smartphones, the details of the gyroscope interface are not included in this report. Based on the user’s winking gesture, six simple and nonoverlapping facial gestures were defined, and a command set was constructed by mapping the corresponding commands. The user’s winking action corresponds to clicking a mouse or touching a screen interface.

### 2.3. Experimental Setup and Sensor Data Collection

**Figure 4:**
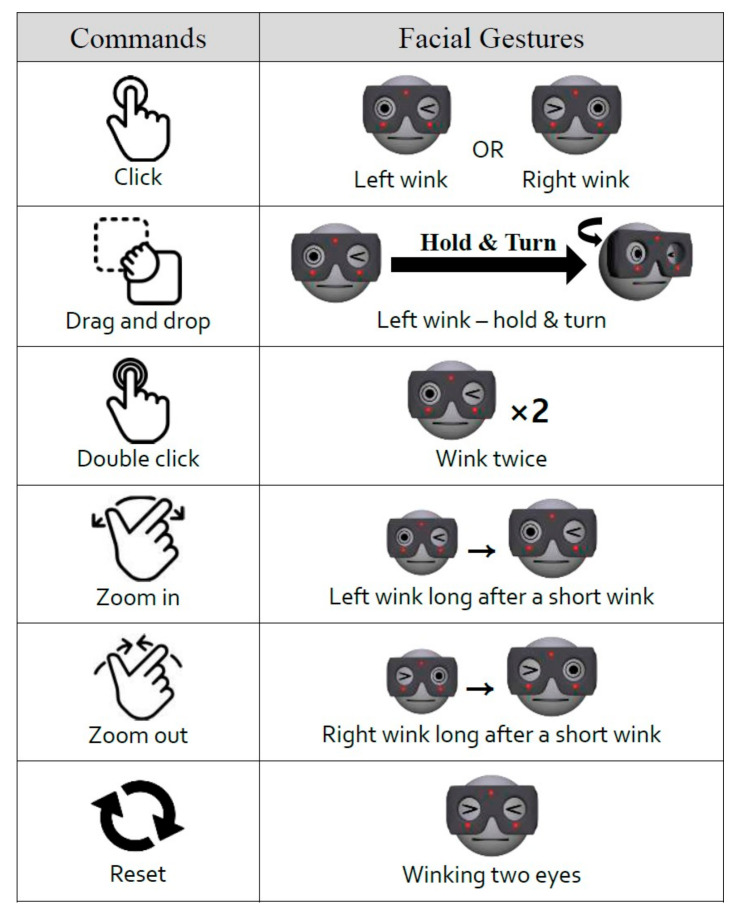
Definition of command set associated with facial gestures and movements. The command set includes control interactions that facilitate a similar user experience compared to the commonly used mouse input devices of PC and touch-screen interfaces of smartphones.

We carried out an experimental validation of the proposed user interface with three groups of volunteers, as follows:

(1) We tested the recognition rate of each command from four participants. In this intensive test, each participant conducted each command 100 times in succession, and we counted the command inputs received from the interface unit.
(2) A user applicability test for 20 participants differed from test 1. In this test, each user randomly performed each command listed in Figure 4 twenty times and counted the number of command inputs received from the interface device.
(3) A usability test was conducted at an exhibition of Information and Communications Technology Forum Korea, (information of the exhibition can be found at the link: https://www.youtube.com/watch?v=8UkGFqAehDI) for more than 100 random visitors, who participated during the exhibition of ICT Forum Korea. The visitors played “move the box” game using prototype VR headset shown in Figure 3. We monitored user feedback on fatigue and inconvenience when playing a game using the prototype VR headset.

**Figure 5:**
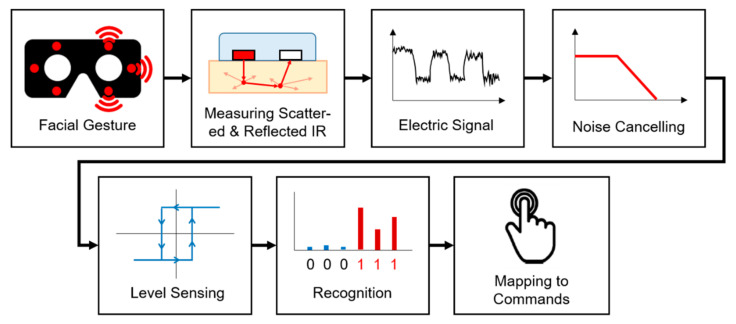
Sensor data calibration process: the IR light emitted into the skin, and the intensity of the reflected IR signals are measured by the IR receiver. Noise is removed by using the low-pass filter of the detected sensor data, and the level is classified into two states by the Schmitt trigger to recognize the user’s command.

We performed a basic experiment to measure the change in sensor value due to facial gestures from experiments involving five participants. Participants consisted of only males aged 22 to 30 years, two of whom were experienced users. The participants wore the prototype and winked instead of clicking a mouse while using a PC. A sampling frequency of 1 kHz was sufficient to recognize instances of winking. Given that different individuals have differently shaped faces, the degree of contact of the sensors is different. As such, the sensor values of the expressionless and gesturing faces were measured prior to the experiment, as a calibration process shown in Figure 5. This process allowed threshold values for the detection of winking to be established.

## 3. Measurements and Results

### 3.1. Physical Layer for Data Acquisition

**Figure 6:**
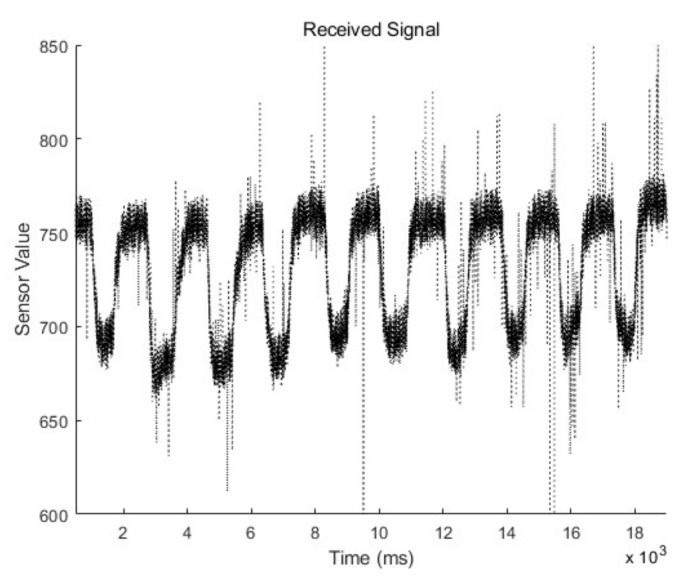
Measured electric signal at location 1 for contact with the skin of the left cheek. The IR receiver measured reflected intensity of scattered IR while the user performs consecutive wink gestures.

When the right eye winks, sensor data from positions 1, 2, and 3 change in accordance with facial skin movement around the right eye; meanwhile, winking the left eye causes a change in the sensor data from positions 4, 5, and 6. There might be a slight time difference among the muscles that contribute to facial gestures, and the received scattered IR values increase or decrease in correspondence with the contraction or relaxation of the skin under the IR coupler. The measured raw data of the electric signal from the IR coupler at location 1 is presented in Figure 6.

By examining the measured data, we found that there is user-to-user variation of the signal intensity. Moreover, the offset value of the electric signal is different depending on the location of the IR coupler, even for the same user. Nevertheless, the intensity and offset values vary for location-to-location and person-to-person, and the shape of the transient signal exhibits certain trends associated with skin movement. Therefore, to adapt the interface to different individuals, the user’s intention must be recognized based on the transient characteristics of the signal, instead of the application of a simple intensity-based threshold approach. In this study, the command type was determined by differentiating the low-pass filtered signal to detect the edge of the transient signal.

According to the data, noise occurs due to uncontrolled skin contact or air gaps between the skin and the IR photocoupler during the contraction and relaxation of the facial muscles. It can be confirmed that the noise generated in the input interface is high-frequency noise, which can be removed by filtering in the digital domain instead of canceling at the device level. Therefore, we used a low-pass filter to preprocess the measured noisy signal.

**Figure 7:**
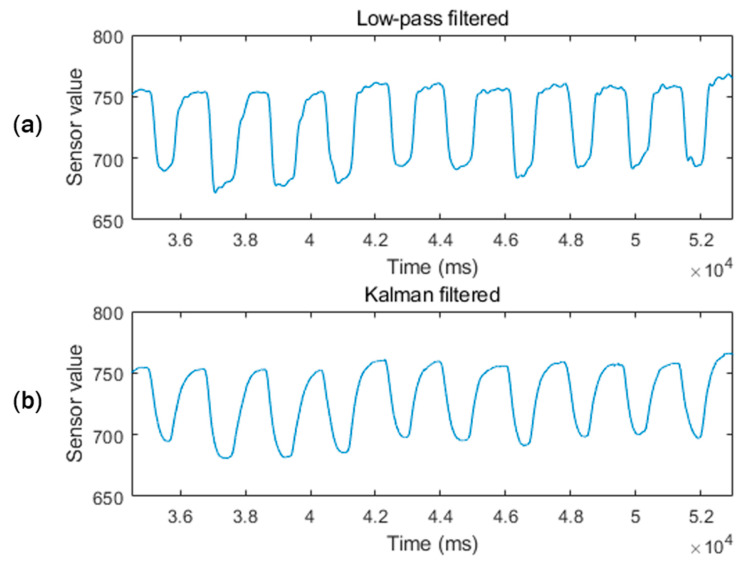
Comparison of noise suppression performance of low-pass and Kalman filters: noise filtering applied to the electric signal shown in Figure 6 using (**a**) a low-pass filter with a sampling frequency Fs=200 Hz and cutoff frequency Fc=0.9 Hz; and (**b**) a Kalman filter with process noise covariance of Q = 10^−5^ and a measurement noise covariance of $R=0.5^{2}$.

**Figure 8:**
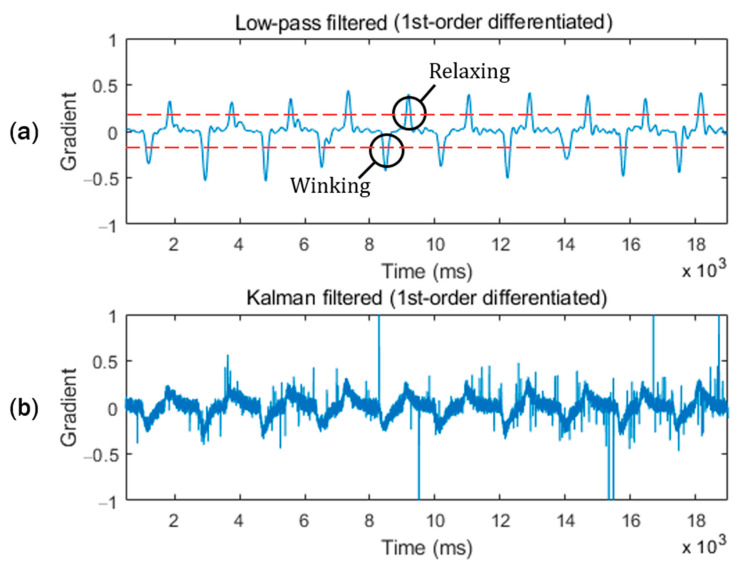
Waveforms of the first-order differentiated signal shown in Figure 7. Figure (**a**) shows that the first derivative of the low-pass filtered signal has 2 distinct valley and peak features for determining the transition to winking gesture and the return to the relaxation posture. (**b**) The first derivative of the Kalman filtered signal becomes very noisy.

Figure 7 shows a comparison of the noise suppression performance of the low-pass and Kalman filters on the measured waveform from the IR coupler at location 1, shown in Figure 6. Both the low-pass filtered and Kalman filtered signals exhibit good noise suppressing performance prior to differentiation of the signal. However, it is not possible to determine the high or low state from the voltage level of the pulse because the offset varies depending on the ambiguous environment of the IR photocoupler on the skin. Therefore, to read the state change, we differentiated the filtered signal to eliminate the effect of the high-frequency noise component. By analyzing the first-order differentiated waveform shown in Figure 8, it was determined that the noise level of the Kalman filtered signal increased. By exploiting the gradient of the low-pass filtered signal, more effective noise cancelation is possible compared to the Kalman filter only.

The first-order derivative of the low-pass filtered signal is sufficient to produce two distinct negative and positive spike-features for determining the transition of the winking gesture and the return to a normal relaxed posture. By applying a simple low-pass filter, the electrical noise measured by the IR photocoupler was sufficiently suppressed to facilitate recognition of facial gestures using the first derivative waveforms.

A Schmitt trigger circuit was utilized to classify the sensor results into two levels: on and off. The threshold of the Schmitt trigger was set based on the first derivative, and the thresholds of the six sensors were set based on each gradient. All participants provided sensor data with consistent trends, which confirms that the proposed method is reliable for the detection of a user’s facial skin movement. The threshold values for detecting a winking pose were empirically set to half of the maximum value of the first derivative of the low-pass filtered signal. We do not need to adjust the decision level for individual face shape, since the threshold value can be adaptively changed to the half of the maximum value of the first derivative of the low-pass filtered signal.

### 3.2. Sensor Data Analysis

**Figure 9:**
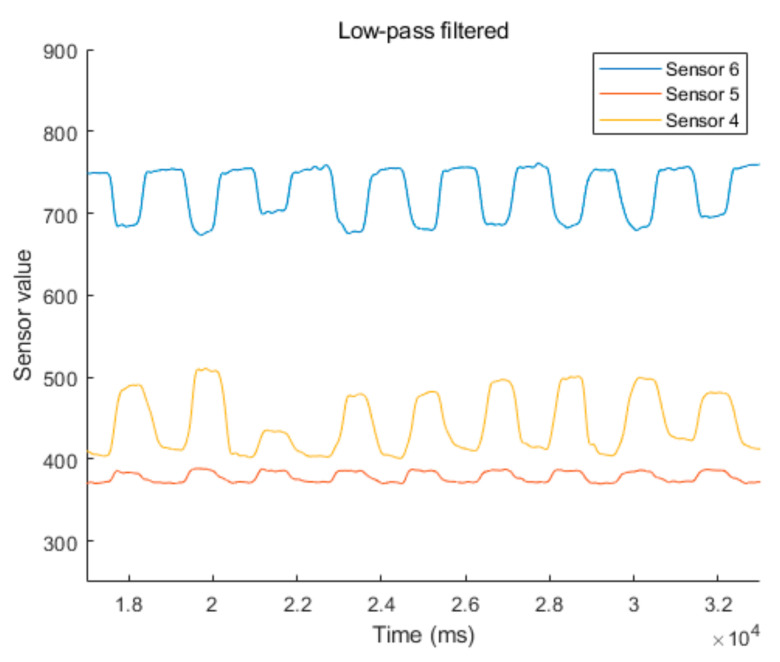
Measured low-pass filtered signals from the sensor positions at 4, 5, and 6 as the user performed consecutive intentional winking processes. The offset values of the measured signals are different for each sensor position.

**Figure 10:**
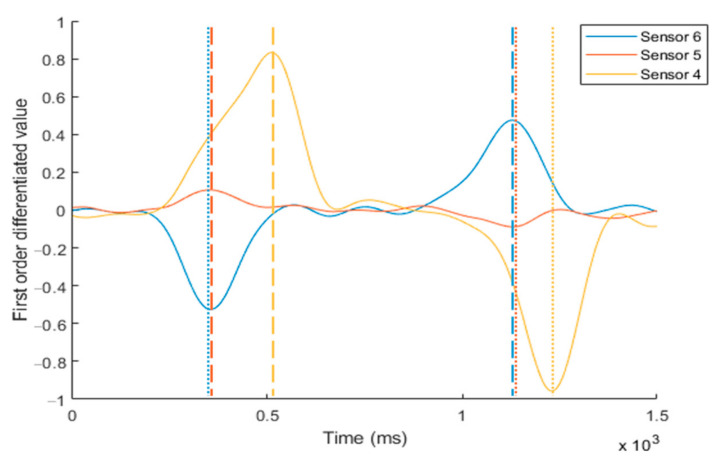
Measured first-order derivative signals from the sensor positions at 4, 5, and 6 during consecutive intentional winking. The time for the peak value of each sensor is not coincident during the winking process. The sequence of muscle movement for the winking process can be measured by tracing the peak points of the signal transition.

In the winking process, the sensor data are synchronized and shifted by a slight time difference, as shown in Figure 9 and Figure 10. First, the muscle near the cheek (sensor 6) moves, and the detected value decreases. Almost simultaneously, the muscle near the temple (sensor 5) moves, and its value increases. The time difference between these two sensor signals was very short. Subsequently, the muscle near the eyebrow (sensor 4) moves, and its value increases. At this point, the time difference is approximately 40–180 ms, depending on the magnitude of winking. As a result, facial gestures are determined by measuring the sequence of the changes in the sensor values in each region of the face. Therefore, it is possible to accurately determine the initial state of the wink, the progression, and the final state. This allows for the characterization of the winking process in humans. The sensor data first increases for approximately 130 ms. When the Schmitt trigger is utilized, the winking process is generally detected within 40–60 ms. This is a reasonable speed for an interface because it is faster than the reaction time of a human operator.

### 3.3. Robustness and Reproducibility of the Proposed Sensing Interface

In the laboratory tests, the interface was tested in two steps. The first experiment was to test the recognition rate of the interface. Four participants made an experiment of performing each facial gesture 100 times, and all participants achieved an accuracy of over 99%, as listed in Table 2. The test subjects were 21- to 29-year-old Asian men, consisting of only inexperienced users. For general user testing, additional experiment was conducted with 20 participants for the evaluation of interface applicability. The test subjects were 21- to 54-year-old Asians; 90% were male and two experienced users were included. As a result of this experiment conducted 20 times for each facial gesture, all participants achieved a 99% success rate. For each experiment, the success rate was measured for the prototype shown in Figure 3.

## 4. Application to Experimental VR Game

To verify the usability as well as to test the user acceptance of the proposed IR photocoupler-based hands-free command set, we developed a VR game with simple tasks that involved moving boxes. The user performed the tasks using the command set click, drag drop, zoom in, and zoom out, as illustrated in Figure 4. Commands could be selected and executed using the prototype HMD.

**Figure 11:**
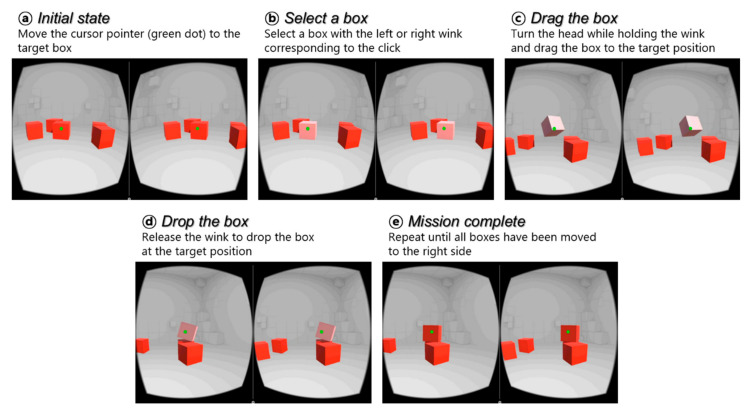
Screenshots from the “move the box” game. The HMD wearer is expected to move boxes located on the left side of the screen to the right using only facial gestures.

**Figure 12:**
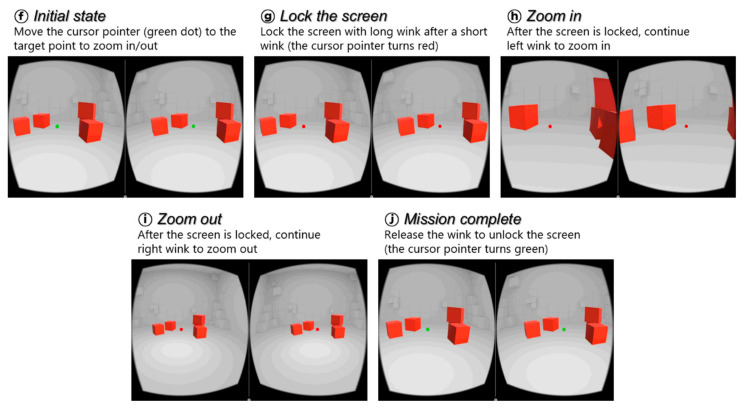
Screenshots from the “zoom-in and zoom-out” game. The user is expected to zoom in and out on the screen using only facial gestures, and then revert to the original size.

**Figure 13:**
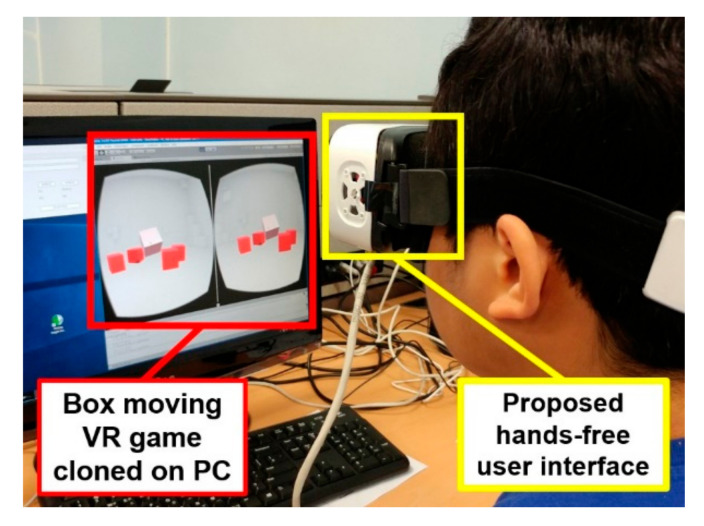
Box-moving game for evaluation of the proposed input interface. To monitor the experiment, the VR game screen was cloned on the PC. The user used facial gestures to move the box in the VR space while performing tasks such as zooming in and out.

In the VR space, there is a cursor at the center of the screen, as represented by the green dot in Figure 11. When the gyroscope built into the VR device detects movement of the head, the screen rotates accordingly, and the cursor corresponding to the center point of the screen also moves. The user moves the cursor to a box and clicks on the box to lift it using a winking gesture. The user must continue the wink gesture while turning his head to move the box and drop it on a target position. The first mission is completed by moving all the boxes to the right side of the screen. In the second mission, the user must select a point on the screen to zoom in and out. When the user places the cursor pointer at a desired position and winks for a long time after a short wink, the cursor pointer turns red, as shown in Figure 12. This state is called the zoom state, which means that the screen is fixed. In the zoom state, if the user continues the left wink gesture, the participant can zoom in on the screen. Otherwise, if the user continues the right wink, the user can zoom out on the screen. The second experiment was completed by restoring the screen to its original size after zooming in and out. Figure 13 shows the experimental setup for the evaluation of the proposed input interface. We uploaded the experimental video on this link, https://www.youtube.com/watch?v=0tl0fiy8fnc.

We evaluated the utility of the proposed user interface compared to a hands-free user interface for VR headsets by conducting an experimental VR game. The experiment was conducted at the exhibition; the age group was distributed from the teens to the 50 s and a large number of both men and women participated. Of the more than 100 random participants, most were inexperienced people who had never even worn VR equipment. We confirmed that the proposed HMD works well for over 95% of participants during usability test at the exhibition of ICT Forum Korea. In the case of young children or women with small faces, the HMD was larger than the face and could not be worn. In very rare cases, some people did not have close contact with the foam interface due to facial asymmetry or severe facial curvature. We need to consider the variation of facial sizes in the design of the foam interface of the headsets.

## 5. Discussion

**Figure 14:**
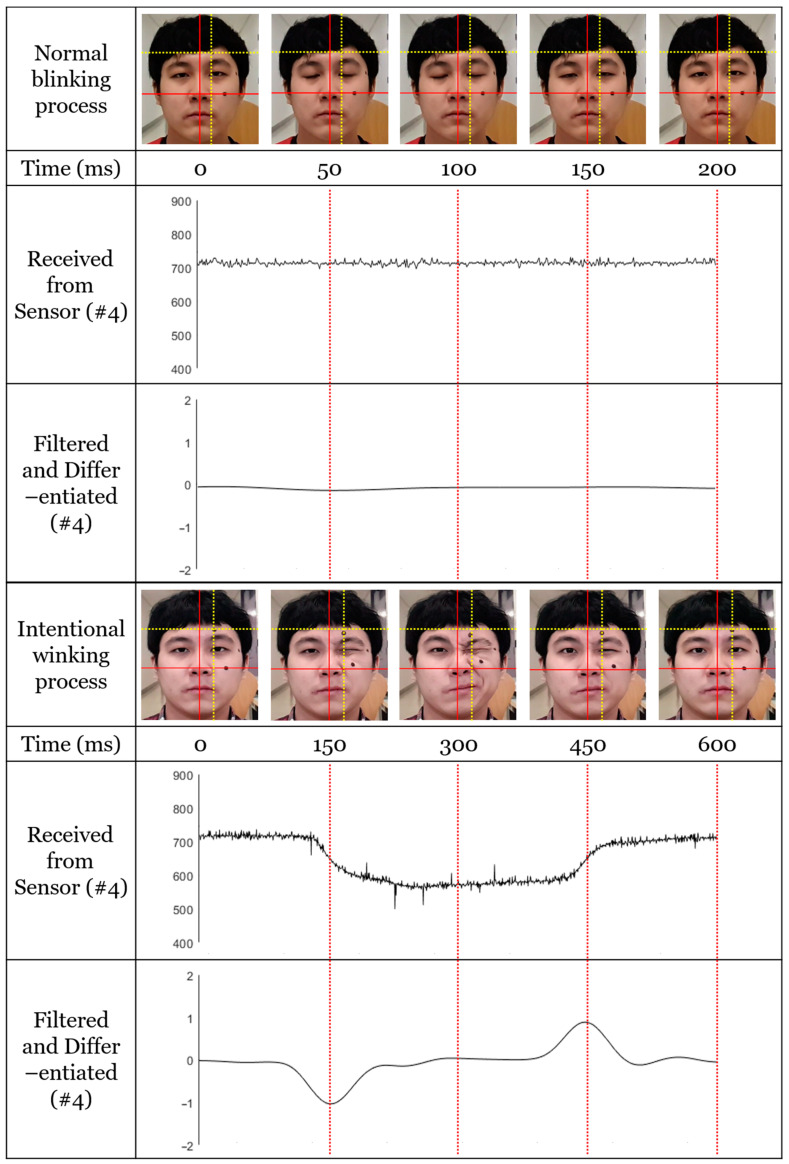
Measured temporal sequence of normal blinking process and intended winking process. The images were acquired using a high-speed camera at 960 fps. The measured typical blinking cycle takes approximately 200 ms, while the intended winking cycle takes approximately 600 ms. The images show that the two marker points on the user’s face did not move during normal blinking; thus, there is no significant transition in the received signal. However, the position of the marker movement is synchronized with temporal transition duration in the received signal in the case of intended winking measurement.

The method must successfully distinguish between the user’s normal blinking and intentional winking gestures. Normal eye blinking was not detected by the IR couplers because the associated skin movement did not cause a significant level of change in the eyebrows, temples, and muscles near the cheeks, i.e., the areas detected by the proposed device. Figure 14 compares the sensor values for left and right intentional eye winking actions and normal blinking actions. In the case of general blinking, the sensor value exhibits only a slight change and no significant signals above the noise level.

To record the movement of the actual muscle, markers were painted in the three areas on the left side of the face (sensors at positions 4, 5, and 6). The position of the nose without facial expression is the coordinate origin, and the marker near the cheek is located at y = 0.

**Figure 15:**
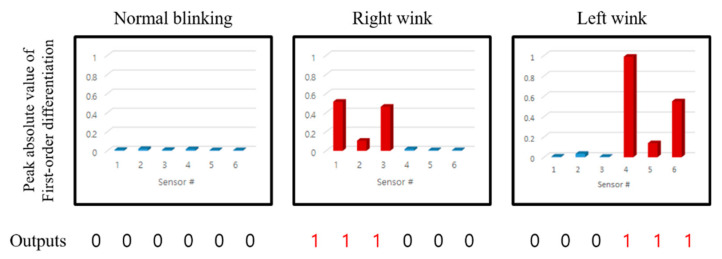
Measured absolute peak value of first order differentiated signal according to sensor positions for normal blinking and intended right/left winking process. The data show that the magnitude of the absolute peak value of first order differentiated signal can be used to detect facial movement caused by intentional winking.

As shown in Figure 14, the muscles (i.e., the cheeks, eyebrows, and temples) do not move; only the eyelids close and open during general blinking. Given that the sensor value does not change significantly, there is no detectable signal above the noise level. In contrast, during intentional winking, the muscles of the cheeks, eyebrows, and temples are mainly used, and the marker of the corresponding area moves significantly, which causes a peak in the sensor value. Figure 15 presents the preprocessed sensor values for the winking action of the left eye; sensors 4, 5, and 6 represent the left eyebrow, temple, and cheek. According to Figure 15, the offset values of the sensors differ for the same person.

The proposed sensor reads the contraction and relaxation of the user’s facial muscles as an intensity change that is measured at a single point based on IR rays traveling through the skin tissue. Given that normal blinking is a semiautomatic function of the eyelid, it causes no significant change in the facial muscles in the six studied areas. Thus, the user’s intentional actions can be distinguished from unintentional actions. In addition, the proposed interface requires only a few resources because the muscle movements can be detected based on the changing sensor values. Thus, this method receives data on the muscle movements around the eyes, which are obscured by the VR headset.

The proposed approach for facial gesture recognition can read the order in which the sensor value of each part changed. Moreover, the results show that the same movement of the facial muscles in the human winking process is recognized in the study. The cheek and eyebrow move during winking. Thus, if the sensor data confirm the sequential movements of the cheek and eyebrow, the movement can be classified as intentional winking. The sensor data of the temple are not essential; however, they can be used as auxiliary data to determine whether intentional winking occurred based on the sensor value.

In the user applicability test among 20 participants, we provided two questionnaires and collected responses from the participants. The questions follow:

(Q1) VR devices may cause motion sickness to users. In your experience, how much did you feel or agree that the headset with facial gesture UI accelerate your motion sickness during experiments compared to conventional HMDs? Please select from 0 (None) to 5 point (Very severely).
(Q2) About the convenience of the user interface, how did you feel while experimenting with the ease of operation? Please select from 0 (Very easy to control) to 5 point (Very difficult to control).

The survey results for Q1 gave a mean = 0.850 and standard deviation = 0.875, and a mean = 0.870 and standard deviation = 0.865 for Q2.

As a result of the participant survey, we could conclude that the proposed device did not accelerate motion sickness for most users, and it could provide an easy-to-control user interface during experiments. Besides the user survey among the 20 participants, we got similar user’s feedback, but oral comments without a survey sheet, from most participants at the exhibition.

In the exhibition test, the results confirm that the proposed user interface works well for various face shapes, regardless of age and gender except for the face that was too small to contact the foam. When the shape of the face changes, the area, and degree of contact of the HMD to the face changes. In addition, facial flexion may change according to the contraction and relaxation of the facial muscles. This can result in a gap between the sensor and the facial skin. However, since this device senses the facial muscle movements of the user, there is only a difference in the intensity of the IR light that enters the skin. Even a small, temporary gap does not affect the sensor values.

Throughout the experiments, we found no participants needing an individual calibration procedure for proper operation. However, since it is a new type of user interface, the first time user needs an acquisition step to learn how to use it before her or his first time usage.

After long-term usage, the proposed device may fail to detect facial gestures under normal operation. First, the original position can change, which alters the detected signal. In this case, since the offset value of the user changed, the movement of the device can be easily recognized, and the device position can be corrected. Second, a significant air gap can occur between the device and the face due to the moving facial muscles. When the sensor is not in close contact with the skin, the peaks of the sensor values are different. The proposed device uses three pairs of sensors to address this problem. Even if the value of one sensor changes, its value can be compensated with that of the corresponding sensors. A subsequent alert reminds the user to wear the device properly.

Some participants had difficulty winking with a specific eye. In this case, the interface can be manufactured for user-specific purposes owing to its excellent variability in the number and positions of the sensors. In addition, because a wink is used as the input, fatigue can occur during prolonged use. The device is designed such that both the left and right eyes can input gestures by winking. Hence, if one eye becomes tired, the other eye can be used. Furthermore, our device is not intended to execute complex commands over a long period of time, but aims to be an auxiliary input device capable of controlling a simple interface in situations in which it is not practical to use the hands. Therefore, the user will not feel tired in a general environment.

In experimental tests, recognition error rate was less than 1.0% for each command associated with facial gestures and movements. All experimental errors were cases where the device did not recognize the user’s intentional facial gestures; nevertheless, there were no error cases misinterpreting the user’s neutral state as a command in the experiments. The experimental results provided us with a trustworthy empirical outcome that the proposed system and method could be applicable to hands-free user interface for VR headsets.

The main objective of proposed system is provide a low-cost auxiliary built-in user interface that can be used in company with other state-of-the-art VR headset controllers for supporting hands-free operations. The proposed user interface was designed in a cost-effective way; the interface hardware is simplified with only six IR photocouplers mounted on the foam, and the filtering processed at the software level. The proposed command set is primarily aimed at providing a user experience similar to a mouse input device on a PC or a touch-screen interface on a smartphone for VR headset users.

## 6. Conclusions

We proposed a facial gesture recognition system based on winking action as a hands-free input method in a VR headset environment. Each facial gesture was mapped one-to-one with the input command, allowing the user’s intention to be translated into an input signal. To read facial gestures, six pairs of IR sensors were attached to the HMD interface foam to detect facial muscle movement. The experiment showed that general blinking and intentional winking could be distinguished, and it was confirmed that a similar change was observed for different people. The operation of the command was tested by playing a simple VR game while wearing a VR headset equipped with the proposed input interface. The proposed user interface cannot replace advanced high fidelity handheld controllers, rather it provides hands-free operation in accompanying other user input devices. Our device is a natural user interface that is completely embedded inside the foam interface and operates only with the user’s wink motion. In addition, our principle is a new input method that is cost-effective compared to the existing hands-free VR interfaces. In addition, it is assumed that a complex and detailed input interface can be realized in the future by combining the proposed technique with various technologies such as gyroscopes, electromyography [26], and voice recognition.

## Figures and Tables

**Table 1:** Survey of interaction methods for the hands-free user interface of VR/augmented reality (AR) headsets.

Modality	Methods	Devices	Accuracy (Mean, %)	Refs
Gaze tracking	Combines traditional gaze-tracking algorithm with geometric model-based convolutional neural network	Eye glass with near-eye viewing device	98.0	[7], 2019
Gaze tracking	Adds extracted feature layers on different receptive fields on top of full preactivation ResNet	Head-mounted display	96.7	[9], 2019
Hand gestures	Real-time gesture recognition exploiting feature descriptors arranged in a multidimensional structure	Head-mounted display	90.0	[10], 2018
Hand gestures	Combines depth and infrared camera streams to enable robust finger-tracking	Head-mounted MR device	96.5	[11], 2018
Hand/hybrid gesture with body motion	Combines motion-based interaction with hand/hybrid gestures for detailed menu selection	Head-mounted AR device	98.1	[12], 2019
Voice recognition	Acoustic model for multi-microphone environment based on the network in network concept with minimum variance distortionless response beamformer for noise reduction	Mobile device	94.2	[13], 2015
Voice recognition	Implements large vocational speech recognition system with small memory, which can be mounted on mobile devices	Mobile device	86.5	[14], 2016
Skin movement	Creates 3D face model in head-mounted display (HMD) environment with eight strain gauges and RGB-D camera	Head-mounted display	NA	[15], 2015
Skin movement	IR-based skin deformation detection with a classifier neural network for spatiotemporal data process	AR glass	95.6	[16], 2019

**Table 2:** Measured recognition rate of each command.

Recognition Rate of Each Command (%)							
User ID	Click	Drag Drop	Double Click	Zoom in	Zoom out	Reset	Average
1	99	100	98	98	99	100	99
2	99	100	98	99	99	99	99
3	100	99	99	100	100	100	99.67
4	99	100	99	99	99	100	99.33
Average	99.25	99.75	98.5	99	99.25	99.75	99.25
